# Professional Registrationists and Others

**Published:** 1917-01-13

**Authors:** 


### THE MATRONS' AND SISTERS' DEPARTMENT.

### PROFESSIONAL REGISTRATIONISTS AND OTHERS.

It is a familiar device of the controversialist to divide the world into his supporters, decked in the white robes of virtue and innocence, and his opponents, whose footwear and clothing hardly hide the cloven hoof and the tail of the Evil One. It is in something of this spirit that the *British Journal of Nursing* has led its readers to contemplate the blameless Registrationists and the sinful Anti-Registrationists, the latter led by that "very unchivalrous knight"—we hardly like to name him, —who has allied himself with the dragon of greed and tyranny, instead of proceeding incontinently to smite off the head of the fell beast bent on the destruction of the Lady, who, in the armour of the "Professional Registrationist," is championing the cause of poor and oppressed nurses throughout the Empire.

The facts are, however, as the Germans say, "quite otherwise." There has been for years an earnest band of Registrationists, how large no one can say—they have, at any rate, been compared to a stage army—but certainly only an insignificant proportion of the Nursing Profession: there has also been an even smaller band of Anti-Registrationists led by Lord Knutsford, and the Medal to be issued in commemoration of his doughty deeds will bear the proud Armada motto, "Dux femina facti." But amid all these alarums and excursions the great bulk of the Nursing Profession has maintained an attitude of absolute indifference, or sturdy neutrality, repelled by the personalities flung by both parties, and not desiring to be spattered by the mud of their high explosives.

As a matter of fact, Lord Knutsford is the champion of a lost cause, and the *British Journal of Nursing* of a cause which will never be won by the methods of assault, which it has hitherto used against its adversaries. In the travesty of the aims and objects of the College of Nursing put forward by the *British Journal of Nursing* last week, it is alleged that its policy is "even more deterrent to sound professional evolution than that of uncompromising obstruction." Surely it is not so many weeks ago that the Central Committee for the State Registration of Nurses, London, was in the thick of very hopeful negotiations for an agreed Bill with the College, negotiations which came only to an end when broken off by the Central Committee for reasons, which most people who read Mr. Stanley's letter and the Committee's statement will regard as wholly inadequate. (See The Hospital, November 18, 1916, pp. 143 to 146.) What has happened in the interval to make the College so much more loathed a thing than it then seemed? Cannot the *Journal* recognise that the time has come to adopt new methods of warfare in attacking the problem of Registration? It is hopeless, in the face of an opposition active enough to divide the House of Commons on the first reading of their Bill, for the Central Committee to continue the warfare on the old lines. Decent folk are disgusted and alienated by the imputation of motives and indiscriminate abuse. They stand aside from those who use such methods of the Hun. They would sooner no Registration than Registration brought about by such means.

The legal recognition of nurses is indeed not a matter that solely concerns the "Professional Registrationist," whatever in this connection the word "professional" may connote. Not only are the rank and file of the profession concerned, but the nurse-training schools, whose curricula must be brought into line, the voluntary hospitals who may have to spend money on class-rooms and equipment, the Poor-Law infirmaries whose probationers may require more time off duty for study, the Local Government Board which has claims that cannot be ignored, the medical profession which employs the nurses, and, finally—to mention no others—the public at large, of whose interests Parliament will prove itself a jealous guardian. We shall be imagining a vain thing if we take our stand upon the exclusive rights and interests of the "Professional Registrationists," and some of us may well be inquiring how the Nurse Secretary of the Central Committee has the right, still to be found "in that galley."

What the much-maligned College has done is to draw on to the side of Registration most, if not all, of those Nursing Schools which in early days were neutral, or, if friendly to the spirit of Registration, disapproved strongly of the methods by which it was sought to be attained. Primarily, the College is based on a voluntary association of the Nursing Schools for the advancement of the Profession of Nursing, and it has placed Legal Registration in the forefront of its programme. It seeks to attain this end by the patient investigation of the many practical difficulties in the way, which it is foolish to ignore and hopeless to override. How is it conceivable that the rank and file of nurses, busy with the honest toil of making a living day by day, and liable at any time to be called from one place to another, can sit week in, week out to solve these problems, even were there any considerable number of them qualified by training and experience to grapple with such complexities? In sober truth, neither matrons nor hospital managers are such ogres as the Editor of the *British Journal of Nursing* is pleased to make them, and there can be few nurses who would not sooner trust their interests to the matrons of the large hospitals and Poor-Law infirmaries, and to women who are experienced in the organisation of nursing, than to a committee of "Professional Registrationists." The spade-work, then, had better be left to such experts in the active exercise of their profession, matrons and others, who are available, not indeed as being perfect instruments and impeccable, but as being more practically knowledgeable and at least as competent as any of the "Professional Registrationists."

What, however, the *British Journal of Nursing* is apt to keep in the background is, that, this nominated Council, though a College Council of which nurses may justly be proud, is but the scaffolding of the completed edifice. That Council will have served its purpose when the great work of registration is well on its way, and the Members of the College can be counted in their thousands; when the Scotch and Irish Boards have reached their full activities; when curricula and examinations are being shaped into uniformity of type and standard; when the clash of competing interests outside is stilled by judicious compromises. Then in 1918—no further off than the middle of next year—one-third of this nominated Council will make way for an equal number of Councillors elected by those nurses who have been wise and patriotic enough to enrol themselves on the Register. Or, as we ourselves confidently believe, before ever that time comes, Parliament will have taken the matter into its own hands, and the Registration Bill of the Royal British College of Nursing will have become law, to the satisfaction of the profession generally.

